# Global methylation correlates with clinical status in multiple sclerosis patients in the first year of IFNbeta treatment

**DOI:** 10.1038/s41598-017-09301-2

**Published:** 2017-08-18

**Authors:** María Jesús Pinto-Medel, Begoña Oliver-Martos, Patricia Urbaneja-Romero, Isaac Hurtado-Guerrero, Jesús Ortega-Pinazo, Pedro Serrano-Castro, Óscar Fernández, Laura Leyva

**Affiliations:** 1 UGC Neurociencias, Laboratorio de Investigación. Instituto de Investigación Biomedica de Málaga (IBIMA), Hospital Regional Universitario de Málaga, Universidad de Málaga (UMA), Málaga, Spain; 2 UGC Neurociencias, Servicio de Neurología, Fundación Pública Andaluza para la Investigación de Málaga en Biomedicina y Salud (FIMABIS), Málaga, Spain; 3 UGC Neurociencias, Servicio de Neurología, Hospital Regional Universitario de Málaga, Málaga, Spain

## Abstract

The alteration of DNA methylation patterns are a key component of disease onset and/or progression. Our objective was to evaluate the differences in Long Interspersed Nuclear Element-1 (LINE-1) methylation levels, as a surrogate marker of global DNA methylation, between multiple sclerosis (MS) patients and healthy controls. In addition, we assessed the association of LINE-1 methylation with clinical disease activity in patients treated with IFNbeta (IFNβ). We found that individuals with high levels of LINE-1 methylation showed 6-fold increased risk of suffering MS. Additionally, treated MS patients who bear high LINE-1 methylation levels had an 11-fold increased risk of clinical activity. Moreover, a negative correlation between treatment duration and percentage of LINE-1 methylation, that was statistically significant exclusively in the group of patients without clinical activity, was observed. Our data suggest that in MS patients, a slight global DNA hypermethylation occurs that may be related to the pathophysiology of the disease. In addition, global DNA methylation levels could play a role as a biomarker for the differential clinical response to IFNβ.

## Introduction

Multiple Sclerosis (MS) is a presumably autoimmune demyelinating and inflammatory chronic disease that targets antigens from the myelin of the central nervous system. Epidemiological studies have reported that MS occurs in genetically predisposed subjects upon whom unknown environmental factors unexpectedly fall. This leads to a failure in the immune response generating an inflammatory process with autoreactive T cells that are able to recognize myelin antigens. After a period of latency, these T cells could be reactivated by a systemic or local factor, leading to an inflammatory response culminating in the destruction of myelin, oligodendrocytes and axons.

IFNbeta (IFNβ) is a first line disease modifying therapy approved for MS treatment. Although numerous randomised clinical trails have demonstrated its beneficial therapeutic effects^1^, the individual patient’s response to this therapy is highly heterogeneous, and a variable percentage of MS patients are considered as non-responders or suboptimal responders to this therapy.

So far, there is not a consensus for classifying responder status and the factors that determine the response to this drug in individual patients have not been fully elucidated, but most therapeutic response criteria classify the patients according to the presence of attacks, a confirmed increase in disability, and active lesions (new in T2 or gadolinium enhancing lesions) in magnetic resonance imaging (MRI), or combinations of these variables after one year’s treatment.

Epigenetics signifies stable and heritable changes in gene expression without changes in the genetic code^2^. Epigenetic mechanisms are responsible for tissue-specific expression^3^ and X-inactivation in female cells^4^. Furthermore, the immune system is tightly regulated at the epigenetic level, associating these epigenetic modifications with various pathological conditions, including cancer, neurological disorders, autoimmune and inflammatory diseases^5^.

One of the major forms of epigenetic marks is DNA methylation^6^. This refers to the covalent addition of a methyl group to the 5′ cytosine located in a CpG site to form 5-methylcytosine (5mC) on the DNA strand^7^. Cytosine methylation is the best understood and most stable epigenetic modification, modulating the transcription of mammalian genomes. CpGs can occur in clusters of higher frequency, known as CpG islands, regions of the genome that are thought to be important in gene regulation, especially when they are localized in the promoter region of a gene. In the human genome, over 70% of CpG sites are methylated at high level (>85%), however CpG islands localized in promoter regions have low methylation levels (<10%)^3, 8^. Generally, CpG islands demonstrate lower methylation levels (hypomethylation) when associated with active genes and higher methylation levels (hypermethylation) when silencing gene expression^7^.

The aberrant gene expression associated with alteration of DNA methylation patterns, is a key component of disease and aging, and has been particularly studied in cancer. The resultant aberrant transcription and chromosomal instability is believed to contribute to disease onset or progression, and increased tumour frequency and malignancy^9–11^. Evidence linking aberrant DNA methylation with idiopathic lupus and drug-induced lupus have been reported^12, 13^. Global decrease of DNA methylation was observed in T cells extracted from patients with active lupus compared with those from normal controls^14, 15^. Mastronardi *et al*. reported the hypomethylation of the peptidylarginine deiminases 2 (PAD2) promoter from MS white matter, and hypothesized that the hypomethylation in normal appearing white matter may be a more general epigenetic phenomenon that may have some biological relevance for gene expression^16^. Other studies have reported differentially methylated CpGs region in CD4^+^ and CD8^+^ T cells associated with MS^17, 18^, with a strong evidence for DNA hypermethylation of CD8+ T cells for MS patients^19^. An altered balance in the expression of DNA methylation and demethylation enzymes has also been reported in relapsing-remitting^20^ and secondary progressive MS patients^21^. In this sense, the latter authors support the idea that hypomethylating agents might have therapeutic effects in MS, as they have already achieved an improvement of the clinical course of experimental autoimmune encephalomyelitis^22^. Conversely, Baranzini *et al*. did not find consistent differences in DNA methylation in a pair of monozygotic twins discordant for MS^23^.

DNA methylation in repetitive elements such as the Long Interspersed Nuclear Element-1 (LINE-1) has been considered a surrogate marker for global genome methylation^24, 25^. LINE-1 is the most abundant family of non-long terminal repeat retrotransposon and constitutes a substantial portion of the human genome (approximately 17%)^26^. It is commonly heavily methylated in normal tissue and the global hypomethylation of DNA is a common event in ageing cells^27^.

Although most epigenetic studies have been focused on cancer, the DNA hypomethylation can contribute to the development of lupus-like diseases and rheumatoid arthritis^28^, but it has to be further studied in other autoimmune diseases.

Accordingly, our aim was to estimate the global DNA methylation in MS patients and healthy controls and its relation with the clinical activity in patients undergoing IFNß therapy.

## Results

### Demographic and clinical characteristics of the patients

The demographic and clinical characteristics of the untreated MS patients and healthy controls are shown in Table 1. All MS patients had relapsing–remitting MS (RRMS). There were no statistically significant differences in gender and age between controls and patients.

**Table 1:** Demographic and clinical characteristics of the untreated MS patients and healthy controls.

	Controls (n = 25)	Non-treated MS patients (n = 54)	p value
Female/Male ratio (% female)	14/11 (56.0%)	36/18 (66.7%)	n.s.
Age in years	34.23 ± 9.83	38.66 ± 9.95	n.s.
Age at onset in years		31.50 ± 11.53	
Disease duration (years)		6.36 ± 8.51	

Date are expressed as mean ± standard deviation unless otherwise stated. n.s.: not significant.

A total of 36 IFNβ-treated RRMS patients were included. Their demographic and clinical characteristics are shown in Table 2.

**Table 2:** Demographic and clinical characteristics of the MS patients treated with IFNβ.

	Treated MS patients (n = 36)		p value
	Without clinical activity (n = 26)	With clinical activity (n = 10)	
Female/Male ratio (% female)	13/13 (50%)	8/2 (80%)	n.s.
Age in years^a^	40.32 ± 7.86	33.60 ± 14.64	n.s.
Age at onset in years^a^	33.15 ± 9.95	25.18 ± 9.78	0.039
Disease duration (years)^a^	7.32 ± 6.55	9.24 ± 6.48	n.s.
Treatment duration (years)^a^	1.23 ± 0.09	1.21 ± 0.10	n.s.
EDSS Score at baseline^b^	1.0 (0.0–1.6)	1.7 (1.0–2.4)	n.s.
EDSS after one year of treatment^b^	1.0 (0.0–1.6)	1.5 (1.0–3.6)	0.038
Relapses in the year prior to treatment^b^	1.0 (0.0–1.0)	1.0 (0.5–1.5)	n.s.
Relapses after one year of treatment^b^	0.0 (0.0–0.0)	1.0 (1.0–1.0)	3.3 × 10^−9^

^a^Data are expressed as mean ± standard deviation unless otherwise stated. ^b^Data are expressed as median (IR); IR: Interquartile Range. EDSS: Expanded Disability Status Scale. n.s.: not significant.

When the clinical activity of the patients during the first year of IFNβ therapy was analyzed, 26 patients had no clinical activity (no relapses and no progression) and 10 showed clinical activity (presence of relapses or an increase of ≥1 point in the EDSS score). There were no statistical differences in gender and age, or in the EDSS score at baseline, or relapses in the year prior to treatment, between both groups. However, age at onset was earlier in those who presented clinical activity.

### Standard curve of methylation analysis

The comparison between the observed and expected values of the standard curve was performed by χ^2^ test. No differences were found between these values at any point in the curve (see Supplementary Fig. S1).

### Association between global DNA methylation and susceptibility to MS

To evaluate the relationship between LINE-1 methylation levels and the risk of developing MS, a logistic regression analysis was performed including untreated MS patients (mean: 65.7; SD: 1.8) and healthy controls (mean: 65.3; SD: 0.8). We considered that a subject presented high levels of LINE-1 methylation when the percentage was higher than or equal to the 75^th^ percentile in the control group (≥65.96%) and conversely, patients with a percentage under the 75^th^ percentile in the control group (<65.96%) were considered as presenting low levels of LINE-1 methylation. Accordingly, 23 untreated patients (42.6%) showed low LINE-1 methylation and 31 (57.4%) high LINE-1 methylation.

In a first step, the association of age and gender with the susceptibility to MS was analyzed, as it is known that age and gender are related to MS, and may be related to the methylation status. We found that, in our first model, age was slightly associated with the risk of MS, including gender as a confounding factor (OR = 1.063; p = 0.035; IC 95% = 1.004–1.125). Subsequently, a logistic regression analysis was performed in a second model including LINE-1 methylation levels with age and gender as potential confounding variables. LINE-1 methylation levels, age and gender showed a statistically significant association with susceptibility to MS (OR = 6.992; p = 0.003; IC 95% = 1.9277–24.763, OR = 1.073; p = 0.021; IC 95% = 1.011–1.139 and OR = 4.456; p = 0.024; IC 95% = 1.222–16.240, respectively) (Table 3).

**Table 3:** Association of LINE-1 methylation levels with risk of MS and clinical activity.

	OR	IC 95%	p value
**Model 1 (risk of MS):**			
Age	1.063	1.004–1.125	0.035
Gender (female *vs*. male)	2.242	0.781–6.438	n.s.
**Model 2 (risk of MS):**			
LINE-1 methylation levels (high *vs*. low)	6.992	1.927–24.763	0.003
Age	1.073	1.011–1.139	0.021
Gender (female *vs*. male)	4.456	1.222–16.240	0.024
**Model 3 (clinical activity):**			
LINE-1 methylation levels (high *vs*. low)	11.046	1.605–76.046	0.015
Age	0.939	0.865–1.020	n.s.
Gender (female *vs*. male)	1.764	0.239–13.02	n.s.

Dependent variable in model 1 and 2: risk of MS (0: control; 1: non-treated MS patients). Dependent variable in model 3: clinical activity status after one year of treatment (0: without clinical activity; 1: with clinical activity). Low LINE-1 methylation: below the 75^th^ percentile in the control group. High LINE-1 methylation: above the 75^th^ percentile in the control group.

**Figure 1:**
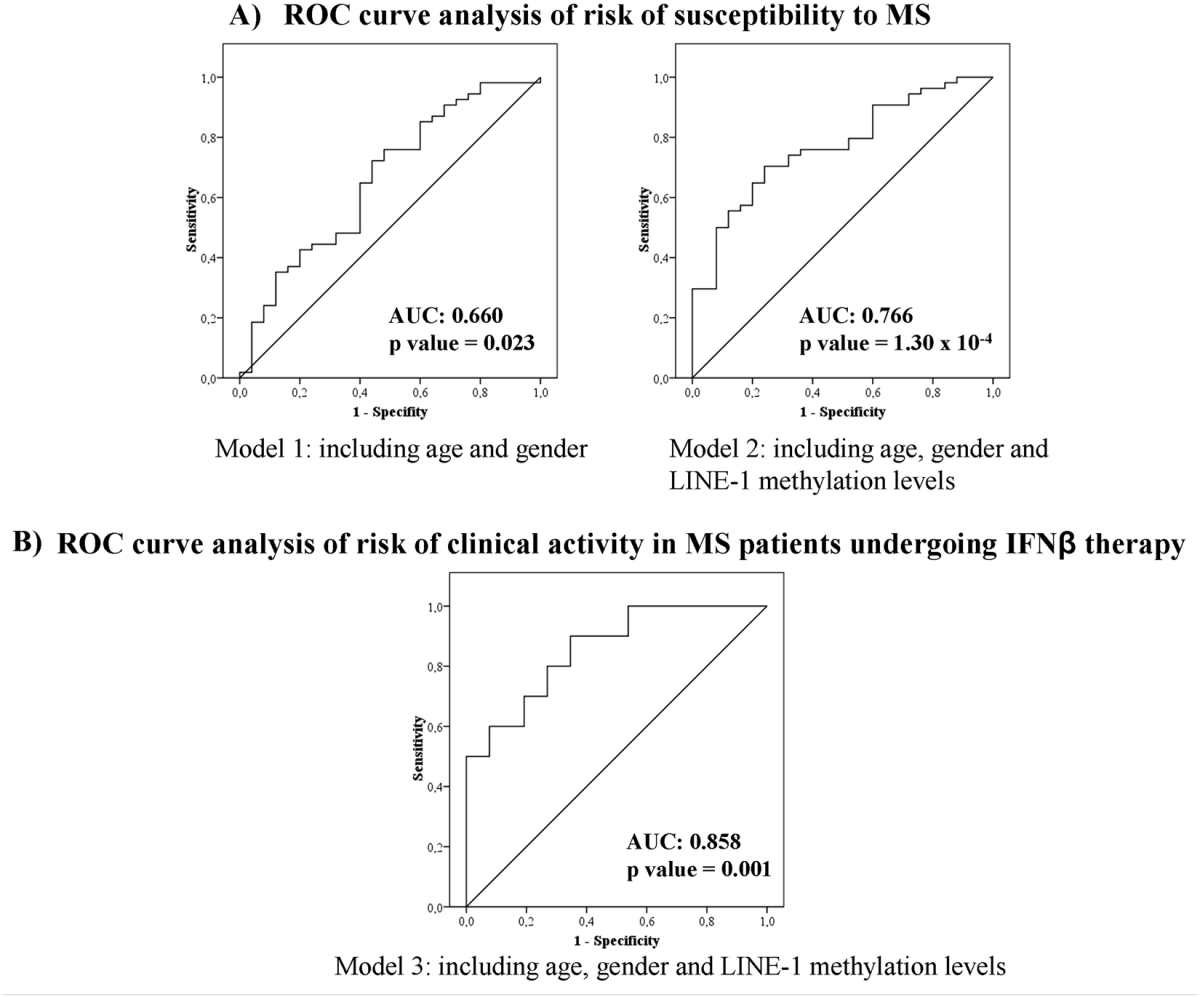
Receiver operating characteristic (ROC) curves for the multivariate logistic regression analysis for the three risk models. Roc curve analysis was performed with the data of probability predicted by the logistic regression models for each individual of the sample, in each of the models. The area under the ROC curve (AUC) is the discriminating power of the performed model. ROC curve analysis of: (**A**) Risk of susceptibility to MS. The predictive capacity was increased when LINE-1 methylation levels were included. The model 2, including LINE-1 methylation levels, had a larger AUC and a higher statistical significance than model 1 including age and gender, exclusively. (**B**) Risk of clinical activity in MS patients undergoing IFNβ therapy. LINE-1 methylation levels had a significant predictive capacity.

Our study showed that subjects with high LINE-1 methylation levels, older ones and females had an increased risk of MS versus subjects with low LINE-1 methylation, younger and males. The areas under the curve (AUC) for the two different risk models were reported to assess the predictive value of LINE-1 methylation in the risk of developing MS. The model 1, that included age and gender, showed an AUC of 0.660 (0.528–0.792) and was statistically significant (p = 0.023). The model 2, also including LINE-1 methylation levels, had a greater AUC of 0.766 (0.663–0.875) and a higher statistical significance (p = 1.30 × 10^−4^). The predictive capacity was increased when LINE-1 methylation was included, as can be observed in Fig. 1A.

### Global DNA methylation and clinical activity in patients undergoing IFNβ therapy

To analyze the relationship between LINE-1 methylation levels and the clinical activity in MS, a logistic regression analysis was performed, including exclusively IFNβ-treated MS patients. In the group of patients without clinical activity during the first year of IFNβ therapy (mean of percentages of LINE-1 methylation: 65.1 SD: 1.3), 19 patients (73.1%) presented low and 7 (26.9%) high LINE-1 methylation levels. In the group of patients with clinical activity despite IFNβ therapy (mean: 66.7 SD: 1.0), 2 patients (20%) showed low LINE-1 methylation percentages whereas 8 (80%) presented high levels. There were not significant differences in LINE-1 methylation according to the type of IFNβ used, as shown in Table 4.

**Table 4:** Contingency table of LINE-1 methylation levels and type of IFNβ.

	LINE- methylation levels	Chi-Square Tests	
**IFNβ used**	**Low**	**High**	n.s.
Rebif	7 (33.3%)	6 (40.0%)	
Betaferon	5 (23.8%)	2 (13.3%)	
Avonex	9 (42.9%)	6 (40.0%)	
Extavia	0 (0.0%)	1 (6.7%)	
Total	21 (100%)	15 (100%)	

In the same way as in the previous analysis, we included LINE-1 methylation levels, age and gender in the logistic regression analysis. Only LINE-1 methylation levels showed a statistically significant association with the clinical activity in patients undergoing IFNβ therapy (OR = 11.046; p = 0.015; IC 95% = 1.605–76.046), and the other two variables were considered confounding factors (model 3 in Table 3). Therefore, treated MS patients with a high percentage of LINE-1 methylation levels had an increased risk of showing clinical activity compared to patients with lower LINE-1 methylation levels.

To asses whether the proposed model that relates LINE-1 methylation levels with the manifestation of clinical activity has a significant predictive capacity, we carried out the ROC curve test. This analysis showed a statistically significant AUC of 0.858 (0.725–0.990), p = 0.001 (Fig. 1B).

**Figure 2:**
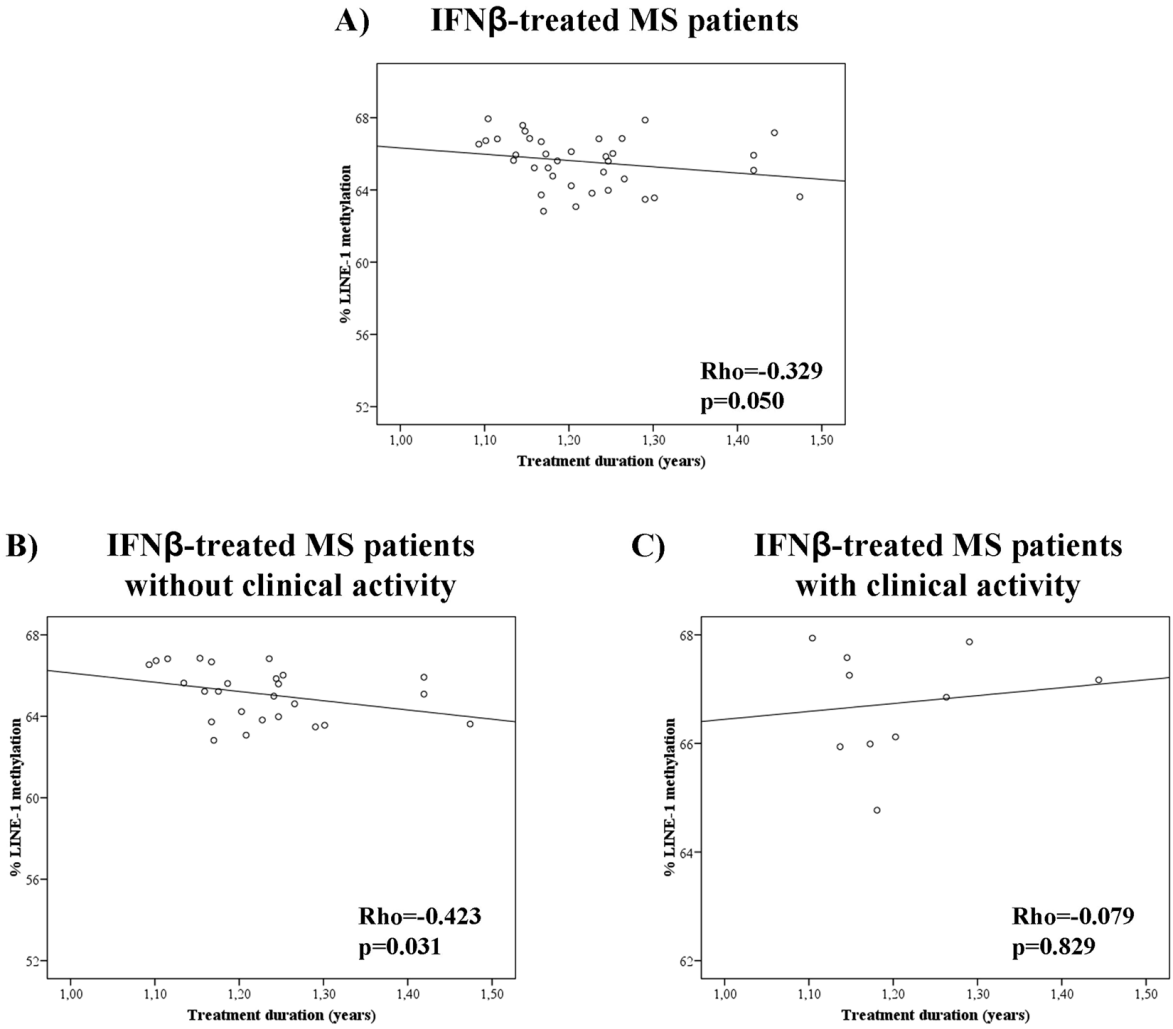
Correlations between the percentage of LINE-1 methylation and IFNβ treatment duration. (**A**) In IFNβ-treated MS patients. (**B**) In treated patients without clinical activity: there was a significant negative correlation between LINE-1 methylation and treatment duration. (**C**) In treated patients with clinical activity there was not a significant correlation.

In addition, we wanted to determine whether the duration of the disease or the treatment duration were related to LINE-1 methylation levels. We observed that disease duration did not significantly correlate to the degree of LINE-1 methylation (data not shown). But when we analyzed the treatment duration, we observed that patients who had been treated for a longer time showed a lower percentage of LINE-1 methylation, although the correlation did not reach statistical significance (Spearman’s Rho = −0.329; p = 0.050) (Fig. 2A). When IFNβ-treated patients were classified according to their clinical activity, in those patients who showed no clinical activity there was a statistically significant negative correlation between treatment duration and percentage of LINE-1 methylation (Spearman’s Rho = −0.423; p = 0.031) (Fig. 2B), so those patients who had longer treatment duration showed a lower percentage of LINE-1 methylation. However this correlation was not observed in patients with clinical activity (Spearman’s Rho = −0.079; p = 0.829) (Fig. 2C).

## Discussion

In the present study, we show a relationship between DNA global methylation levels in PBMC and MS. In epigenetic wide association studies it has been shown that, in neurological diseases, there is a concordance between methylation levels of peripheral blood and brain, allowing studies in PBMC to provide valuable information about the pathology of these diseases^29^.

Although the mean percentage of methylation of LINE-1 remains relatively stable^30^, it appears to be affected by a variety of environmental factors^31^. LINE-1 DNA sequences are repeated throughout the genome, and those with intact 5′ promoters can copy and insert themselves elsewhere in the cellular DNA^32^, potentially affecting gene expression^33^ and may result in disease^34^.

Some authors have reported LINE-1 hypomethylation in different diseases, such as lung cancer^35^, systemic lupus erythematosus^36^, rheumatoid arthritis^37, 38^ or cardiovascular disease^39^. Other studies have shown global DNA hypermethylation in cutaneous melanoma^40^ or neurodegenerative and neurological disorders^41, 42^. We found a risk of susceptibility to MS of 6.9-fold associated with high LINE-1 methylation levels in PBMC, suggesting that the genome of MS patients show a higher degree of methylation that could be associated with a higher frequency of chromosomal aberrations^43^ and an increased DNA damage^44^.

Additionally, we found that IFNβ treated MS patients with high LINE-1 methylation levels had an 11-fold increased risk of developing clinical activity during treatment. This result was supported by the significant negative correlation between treatment duration and LINE-1 methylation levels, in treated patients without clinical activity and not in patients with clinical activity. This fact showed us that IFNβ therapy was able to decrease significantly LINE-1 methylation levels in MS patients without clinical activity but hypermethylation was maintained at the same levels as before the onset of therapy in patients in whom the treatment was not effective in decreasing clinical activity.One explanation could be that, as Searles Nielsen *et al*. reported, the persons with less ability to respond to a changing environment may be at increased risk of developing a variety of neurological disorders^45^. In this case, the patients with less ability to respond to the IFNβ therapy showed an increased risk of developing clinical activity. The decrease of LINE-1 methylation levels in MS patients without clinical activity could be related to an increased expression profile of genes associated with IFNβ response, such as those involved in the upregulation of production of antiinflammatory mediators such as TGF-β1, IL1ra, IL6 and IL10^46–48^. In addition, this significant decrease in global methylation levels could be related both to an increase in the percentage of Treg cells (CD4^+^CD25^+^FoxP3^+^) and to the restoration of the suppressive function of these cells, described in MS patients treated with IFNβ^49^, since Mangano *et al*. also reported both an increase in the percentage of Treg cells and a restoration of their function in an EAE model after treatment with the hypomethylating agent (5-aza-2′-deoxycytidine), by inducing Foxp3 expression via demethylation of a CpG island in Foxp3^22^.

Assessing PBMC DNA methylation has the potential to be a highly cost-effective and tissue accessible approach to population screening, in order to identify those patients under treatment with an increased risk of developing clinical activity.

The differences in LINE-1 methylation found among different groups may seem very small, but DNA methylation levels determined by pyrosequencing technology are highly reproducible and accurate to quantify DNA methylation. Additionally, small differences in LINE-1 methylation have been associated with the risk of cancer related to exposure to environmental determinants^50^.

Our data suggest that in MS patients a slight global DNA hypermethylation occurs which may be related to the pathophysiology of the disease. Furthermore, this has been supported by the association between the absence of clinical activity and the decrease of this hypermethylation that we have reported.

More studies will be necessary in the future to establish the relationship between epigenetic events and MS or its treatment.

## Methods

### Subjects

A total of 95 Caucasian patients with clinically definite MS, according to the McDonald criteria^51^, were recruited through the Multiple Sclerosis Unit of the Hospital Regional Universitario de Malaga, in Spain. Fifty four patients were treatment-naïve and the remaining 41 were undergoing treatment with any of the commercially available IFN-β molecules for over a year. None of the patients had received corticosteroids for at least 3 months before enrolment. As controls, 25 healthy unrelated subjects were obtained from the BioBank of our Hospital.

Samples were processed following standard procedures immediately after their reception. Genomic DNA was isolated from peripheral blood nucleated cells using the Puregene DNA isolation kit (Gentra Systems, MN, USA), according to the manufacturer’s protocol. DNA samples were frozen by the Malaga Regional Hospital Biobank, as part of Andalusian Public Health System Biobank. All patients participating in the study gave their informed consent and protocols were approved by the Institutional Research Ethics Committee (Comisión de Etica y de Investigación del Hospital Regional Universitario de Malaga). All experiments were performed in accordance with relevant guidelines and regulations.

The criteria to classify patients with clinical activity during the first year of IFN-β treatment were the presence of any relapses or a confirmed increase at 6 months of at least one point in the EDSS score. Active lesions in MRI have not been included in our criteria as, unfortunately, MRI at the onset of therapy and after one year’s treatment are not always available for all the patients in our hospital.

The presence of neutralizing antibodies against IFNβ in serum in MS patients treated with IFNβ was determined by the cytopathic effect test, following the recommendations of The World Health Organization (WHO)^52^. Five patients had low titres of neutralizing antibodies against IFNβ (20–80 TRU) and were excluded from the study.

### DNA Methylation

Briefly, 1.5 μg of DNA was bisulfite treated with the EpiTect bisulfite kit (Qiagen, Hilden, Germany) as recommended by the manufacturer, that converts non-methylated cytosines to uracils while leaving methylated cytosines unmodified. The DNA was re-suspended in 20 μl of Buffer EB (supplied in the kit) and stored at −20 °C until further use.

Bisulfite treated samples were used to assess DNA methylation of the LINE-1 repetitive element as a surrogate marker for global DNA methylation changes. PCR was carried out in a 25 μl reaction mix containing 150ng bisulfite-converted DNA, 1x Pyromark PCR Master Mix (Qiagen), 1x Coral Load Concentrate (Qiagen) and a final primer concentration of 0.2 μM. One of the primers was biotinylated in order to purify the final PCR product using Streptavidin Sepharose HP beads. The PCR program used was 95 °C for 15 minutes, then 45 cycles of 94 °C for 30 seconds followed by 58 °C for 30 seconds and 72 °C for 30 seconds, with a final extension at 72 °C for 10 minutes. The amplified PCR product of 100 bp included 6 CpG sites.

The biotinylated PCR products were purified and converted into single-strands to act as a template in the pyrosequencing reaction using the pyrosequencing Vacuum PrepTool (Qiagen).

The PyroMark^TM^Q96 ID Pyrosequencing System (Qiagen) was used to determine the methylation status of the CpG island region of the LINE-1 element of all samples in duplicate. The pyrosequencing involves the stepwise incorporation of deoxynucleotide triphosphates into the growing strand of nascent DNA. For this, 20 μl of the PCR products underwent pyrosequencing using a 0.4 μM sequencing primer. The primer sequences used in the PCR and pyrosequencing reactions were previously published by Daskalos *et al*.^53^.

Non-CpG cytosine residues were used as internal controls to verify efficient sodium bisulfite DNA conversion. The control peak for the bisulphite conversion passed the quality assessment for all the samples. Jurkat Genomic and CpG Methylated Jurkat Genomic DNA (New England BioLabs, Ipswich, MA) were used as experimental controls in each run.

Methylation data were analysed using PyroMark CpG software (Qiagen). The degree of methylation was expressed as a percentage of methylated cytosines over the sum of methylated and unmethylated cytosines. The mean methylation for the 6 CpG sites was used as the measure of global DNA methylation of LINE-1. The results that had no signal or a low signal were excluded.

LINE-1 methylation was measured in duplicate samples and the average coefficient variation was <0.8%. To calculate inter-assay variability the same two samples were run in duplicate in all the analyzed plates, and the coefficient variation was <2.0%.

### Standard curve of methylation

Jurkat Genomic and CpG Methylated Jurkat Genomic DNA (New England BioLabs), previously bisulphite treated, were mixed in different ratios to obtain calibration samples that represent distinct methylation percentages of 60.40%, 64.50%, 64.84%, 65.19%, 65.52%, 65.86%, 66.55%, 67.72% and 68.60%. Jurkat Genomic and CpG Methylated Jurkat Genomic DNA have 60.4% and 68.6% of LINE-1 methylation respectively.

### Statistical analysis

Descriptive statistics show means and standard deviations or median and inter-quartile ranges for quantitative variables and proportions for categorical variables.

Normal distribution of all variables was tested by the Kolmogorov-Smirnov test. The statistical differences between the means of quantitative variables were tested using the one-way ANOVA or T-student test for parametric variables and the Mann-Whitney test for non-parametric variables. The assessment of qualitative variables was performed by means of a χ^2^. The correlation between levels of LINE-1 methylation and demographic and clinical variables was assessed using Spearman’s correlation coefficients.

The strength of association between variables was measured by calculating the odds ratio (OR) and 95% confidence intervals by multivariate logistic regression analysis, controlled for potential confounders such as age and gender. The level of LINE-1 methylation was categorized into two categories corresponding to low LINE-1 methylation (below the 75^th^ percentile found in the control group) and high LINE-1 methylation (above the 75^th^ percentile in the control group).

Receiver Operating Characteristic (ROC)^54^ generated for the multivariate logistic regression analysis and quantified using the areas under the curve (AUC) were performed to evaluate the predictive value of LINE-1 methylation levels in the diagnosis of MS or on the clinical activity in patients undergoing IFNβ therapy.

All tests were two-sided and p < 0.05 was considered to be statistically significant. All analyses were performed using SPSS 15.0 statistical software.

## Electronic supplementary material

Supplementary Information
