# Supplementary material for: Global methylation correlates with clinical status in multiple sclerosis patients in the first year of IFNbeta treatment
Source: Sci Rep. 2017 Aug 18;7:8727. doi: 10.1038/s41598-017-09301-2 (PMC5562733; doi:10.1038/s41598-017-09301-2)
Supplement: Supplementary file 1 — Supplementary Information [file 41598_2017_9301_MOESM1_ESM.pdf]

Global methylation correlates with clinical status in multiple sclerosis patients in the first year of IFNbeta treatment

María Jesús Pinto-Medel*, Begoña Oliver-Martos, Patricia Urbaneja-Romero, Isaac Hurtado-Guerrero, Jesús Ortega-Pinazo, Pedro Serrano-Castro, Óscar Fernández, Laura Leyva Fernández

Supplementary Fig. S1: Standard curve of global DNA methylation.

Expected values	Observed values	X2=(O-E)^2/E	
60.40	60.36	3.35 x 10^{-5}	n.s
64.50	63.42	1.82 x 10^{-2}	n.s
64.84	62.72	6.93 x 10^{-2}	n.s
65.19	63.79	3.03 x 10^{-2}	n.s
65.52	65.54	6.10 x 10^{-6}	n.s
65.86	64.94	1.30 x 10^{-2}	n.s
66.55	68.78	7.47 x 10^{-2}	n.s
67.72	65.53	7.08 x 10-2	n.s
68.60	68.58	9.11 x 10^{-6}	n.s

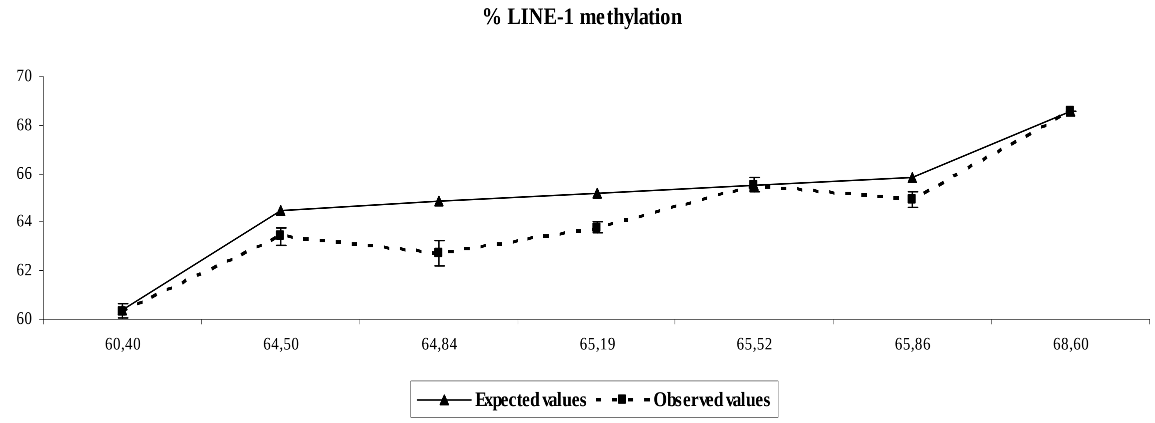

Values observed and expected for every point of the curve.
